# Microsatellite Phenotype as a Guide for Immunotherapy in Colorectal Cancer: Current Status and Future Perspectives

**DOI:** 10.3390/genes17060674

**Published:** 2026-06-09

**Authors:** Evangelos Koustas, Eleni-Myrto Trifylli, Vaios Oraiopoulos, Michalis V. Karamouzis, Panagiotis Sarantis

**Affiliations:** 1 Oncology Department, General Hospital Evangelismos, Ipsilantou 45-47, 106 76 Athens, Greece; 2 Institute of Molecular Medicine and Biomedical Research, 115 27 Athens, Greece; trif.lena@gmail.com (E.-M.T.); mkaramouz@med.uoa.gr (M.V.K.); panayotissarantis@gmail.com (P.S.); 3 University Clinic of Internal Medicine, General and Oncology Hospital “Agioi Anargyroi”, National and Kapodistrian University of Athens, Timiou Stavrou 14, 145 64 Kifisia, Greece; oraiopoulosv@gmail.com

**Keywords:** colorectal cancer, checkpoint inhibitors, immunotherapy, microsatellite instability

## Abstract

The therapeutic armamentarium for colorectal cancer (CRC) has been significantly expanded with the introduction of immunotherapy, particularly immune checkpoint inhibitors (ICIs). However, the response to immunotherapy is strongly dependent on microsatellite instability (MSI) status. Tumors with high MSI (MSI-H) and/or mismatch repair deficiency (dMMR) exhibit high tumor mutational burden (TMB), increased neoantigen load, and enhanced immunogenicity, leading to improved responses to ICIs compared with microsatellite-stable (MSS) and/or mismatch repair-proficient (pMMR) tumors. This has changed the treatment landscape of this small subgroup of metastatic CRC (mCRC), including the approval of pembrolizumab as a first-line option. In contrast, most mCRC cases are MSS/pMMR and are resistant or poorly responsive to ICIs, with no established standard immunotherapy strategy. Therefore, current approaches aim to convert these “cold” tumors into “hot,” immunologically active tumors. This review summarizes the distinct molecular basis of MSI phenotypes and their interaction with the tumor microenvironment, and provides relevant insights into current clinical evidence for prognostic and predictive biomarkers beyond MSI status, as well as novel therapeutic strategies to overcome resistance in MSS disease.

## 1. Introduction

Colorectal cancer (CRC) represents a major global health burden and remains one of the leading causes of cancer-related mortality worldwide. Despite substantial advances in drug development and therapeutic strategies, it still poses a clinical challenge [1]. Notwithstanding significant improvements in clinical outcomes, as well as advances in diagnostic strategies (including screening) and therapeutic modalities, such as surgery, chemotherapy, targeted therapy, and, more recently, immunotherapy, metastatic CRC continues to be associated with substantial morbidity and mortality [2].

A major advance in tailoring immunotherapy for CRC management has been the recognition that microsatellite phenotype, which is broadly categorized as MSI-H and/or dMMR versus MSS and/or pMMR, is a strong predictor of responsiveness to immunotherapy. MSI/MMR phenotype testing has now become a gold standard in the evaluation of CRC patients, as it provides important information regarding prognosis, familial risk (including conditions such as Lynch syndrome), and therapeutic decision-making [3].

Microsatellites are short, tandemly repeated DNA sequences that are scattered throughout the genome [4]. Slippage events at microsatellites generate insertion/deletion loops during DNA replication, while mismatch repair (MMR) machinery (MLH1, MSH2, MSH6, and PMS2) normally corrects these errors. However, the loss of the mismatch repair (MMR) function, which may occur due to somatic mutations or germline alterations, as well as epigenetic silencing (MLH1 promoter hypermethylation), leads to deficient MMR (dMMR) and microsatellite instability-high (MSI-H) phenotype [5]. Moreover, the genetic consequences of dMMR include the accumulation of frameshift mutations across coding sequences and the generation of numerous novel frameshift-derived peptides (neoantigens) [6,7]. The total number of somatic mutations present within the coding regions of a tumor genome is described by the term Tumor mutational burden (TMB) [8]. Elevated TMB has been associated with increased tumor immunogenicity and improved responsiveness to ICIs in several tumor types, primarily through neoantigen generation that can be recognized by the anti-tumor immune system [9,10]. However, the predictive value of TMB in CRC remains context-dependent and is influenced by several factors such as cause of hypermutation, microsatellite status, and the biological background of the tumor [9,10].

The high production of these neoantigens increases tumor immunogenicity and promotes the recruitment of tumor-infiltrating lymphocytes (TILs), resulting in an “inflamed” tumor microenvironment. In this context, immune activation is often accompanied by compensatory upregulation of immune checkpoint pathways, including the PD-1/PD-L1 axis and CTLA-4, as a mechanism of adaptive immune resistance. In contrast, pMMR/MSS tumors typically exhibit lower tumor mutational burden (TMB) and fewer neoantigens, which leads to reduced tumor immunogenicity, a non-inflamed (“cold”) microenvironment, and consequently greater primary resistance to immune checkpoint inhibitors [11].

Furthermore, less than 15% of all CRC patients display MSI-H phenotype, but MSI-H/dMMR is far more common in the early stage of the disease than in the metastatic stage (which comprises roughly 3–5% of cases). MSI-H tumors are disproportionately located in the proximal colon and are more likely to show poor differentiation, mucinous histology, and abundant lymphocytic infiltrate [12]. A subset of MSI-H CRC is due to (i) Lynch syndrome, an inherited genetic disorder caused by pathogenic variants in DNA mismatch repair genes (germline pathogenic variants in MMR genes), which accounts for a minority but clinically important portion of MSI-H cases, while the remainder are (ii) sporadic, frequently related to MLH1 promoter hypermethylation often associated with BRAF V600E mutation (8–10% of metastatic colorectal cancer (mCRC) patients). It has been underlined that the sporadicity of mutant BRAFV600E almost certainly indicates sporadic MSI-H/dMMR [5]. The former subset is not typically associated with BRAFV600E mutations, whereas the latter is. On top of that, sporadic MSI-H CRC is correlated with the hypermethylator phenotype CIMP (CpG island methylator phenotype), which also gives rise to the MSI-H phenotype, as the MLH1 promoter is hypermethylated [6]. Both of these entities are chemoresistant, and they are associated with poor survival and outcomes, with or without the administration of targeted therapy.

Based on the aforementioned evidence, universal MSI/MMR testing is considered pivotal for patients with CRC, not only for Lynch syndrome screening but also for prognostic stratification and immunotherapy selection, as the distinction between MSI-H/dMMR and MSS/pMMR tumors is fundamental to understanding the differential responses to immunotherapy in CRC [13]. This review provides an overview of the molecular background of MSI phenotypes and their role in tumor–immune interactions in colorectal cancer. It evaluates current clinical evidence, discusses combinatorial therapeutic strategies for MSS disease, identifies potential biomarkers beyond MSI-H status, and addresses mechanisms of primary and acquired resistance with practical insights for clinicians and researchers.

## 2. TME Alterations Across Microsatellite Phenotypes and Immunotherapy Resistance Mechanisms

MSI-H/dMMR and MSS/pMMR CRCs exhibit distinct biological and immunological profiles, leading to different mechanisms of immune evasion and characteristic tumor microenvironment (TME) alterations. These differences are fundamental in shaping responses to immunotherapy and guiding rational therapeutic strategies.

### 2.1. MSI-H/dMMR CRC

CRC tumors with the MSI-H/dMMR phenotype typically exhibit three key molecular characteristics: (i) an elevated tumor mutational burden (TMB) resulting from defective DNA mismatch repair; (ii) an increased neoantigen load, comprising novel peptides generated by tumor-specific genetic mutations, which enhance immune recognition and promote CD8^+^ T-cell infiltration [14]; and (iii) a dense immune infiltrate, including natural killer (NK) cells, CD8^+^ cytotoxic T cells, and Th1 cells, contributing to effective antitumor immune activity. Meanwhile, MSI-H/dMMR CRC tumors are characterized by increased production of pro-inflammatory cytokines, including IFN-γ, CXCL9, and CXCL10, which are associated with activation of the IFN-γ signaling pathway and further facilitate the recruitment and activation of cytotoxic T lymphocytes within the tumor microenvironment [14]. Moreover, another key feature of these tumors is (iv) the overexpression of immune checkpoint molecules (e.g., PD-1, PD-L1, CTLA-4), indicating an adaptive immune resistance phenotype that can be reversed by the administration of ICIs, such as pembrolizumab and nivolumab, presenting a higher responsiveness to them [15,16].

#### 2.1.1. Primary or Acquired Immunotherapy Resistance in MSI-H/dMMR CRC Tumors

Even among CRC patients with MSI-H who initially respond to checkpoint inhibitors, some relapse due to acquired resistance. Several primary and acquired resistance mechanisms have been discovered.

#### 2.1.2. Loss of Antigen Presentation

Mutations in β2-microglobulin or HLA genes can impair neoantigen presentation by disrupting the antigen presentation machinery (APM). The APM primarily consists of Major Histocompatibility Complex class I and II molecules (MHC-I and MHC-II) along with associated components, including β2-microglobulin (B2M). Effective recognition of cancer cells by the immune system depends on the presentation of non-self-peptides (neoantigens) by tumor cells through MHC class I or II complexes. When MHC molecules or their associated subunits are lost or downregulated, T-cell-mediated anti-tumor immune responses can be significantly weakened [17]. Disruption of peptide loading onto MHC molecules significantly impairs neoantigen presentation and its recognition by CD8^+^ T cells, despite the high neoantigen burden [14,15,16]. On top of that, defects in the IFN-γ signaling pathway, such as JAK1/JAK2 mutations, alter MHC expression and weaken the antineoplastic immune response by limiting T-cell-mediated cytotoxicity.

Moreover, tumor heterogeneity contributes to the emergence of cancer cell clones with reduced neoantigen expression or mutations that diminish immunogenicity. Only a limited proportion of tumor mutations produce genuinely immunogenic neoantigens, allowing tumors to gradually evolve toward lower immunogenic potential. Estimates suggest that fewer than 5% of tumor mutations generate peptides capable of eliciting strong immune responses, as many mutated peptides closely resemble normal proteins and therefore fail to trigger effective immune recognition. In addition, immune pressure may promote immunoediting, a process in which highly immunogenic tumor clones are eliminated while those with fewer or less immunogenic neoantigens persist and proliferate. This evolutionary selection facilitates immune escape and may reduce the effectiveness of immune checkpoint inhibitors (ICIs) [12,13,14,15,16,18].

#### 2.1.3. Upregulation/Compensatory Induction of Alternate Inhibitory Receptors and Oncogenic Signaling Pathway

In CRC patients, chronic antigen exposure within the TME leads to T-cell exhaustion, which is characterized by increased expression of inhibitory immune checkpoint molecules such as TIM-3 and LAG-3, together with immunosuppressive cytokines that inhibit effector T-cell function and lead to resistance to ICI [19]. Moreover, activation of well-known signaling pathways such as RAS/RAF/MEK/ERK and PI3K/AKT/mTOR contributes to tumor progression and immune evasion by promoting tumor cell proliferation, reducing neoantigen presentation, and augmenting an immunosuppressive TME [20].

#### 2.1.4. Tumor Microenvironment (TME) Remodeling

Several studies have shown alterations in the cellular components of TME, including increased infiltration of immunosuppressive cell populations such as myeloid-derived suppressor cells (MDSCs), regulatory T cells (Tregs), and tumor-associated macrophages (TAMs) with an M2 phenotype. These immunosuppressive cells are responsible for inhibiting cytotoxic T-cell activity and facilitating tumor immune evasion [21]. Understanding these mechanisms informs rational combinations (e.g., dual checkpoint blockade to target alternative inhibitory receptors, agents to restore antigen presentation, or therapies to reprogram suppressive myeloid cells). In addition, T-cell functionality is further reduced under hypoxic conditions or in the presence of excessive lactate production by tumor cells.

### 2.2. MSS/pMMR CRC Phenotype and Resistance Mechanisms

Approximately 95% of metastatic CRC cases are MSS/pMMR, whereas MSI-H/dMMR tumors account for only 3–5% of metastatic disease.

Compared with the MSI-H/dMMR phenotype, MSS/pMMR tumors frequently exhibit (i) low TMB and (ii) a reduced neoantigen load, which renders malignant cells less visible to cytotoxic T cells due to their limited immunogenicity. More specifically, impaired antigen presentation further contributes to the immune system’s inability to recognize and eliminate tumor cells. This is partly attributed to low TMB, which generates fewer neoantigens capable of eliciting a strong cytotoxic response, and to defects in the antigen-processing machinery, including reduced expression and/or functionality of the MHC class I complex, ultimately resulting in inadequate antitumor immune activation and immune escape [16,17,18,19,20,21,22].

Another characteristic feature of this phenotype is a TME marked by (iii) scant infiltration of effector T cells and (iv) enrichment of immunosuppressive populations, including Tregs, myeloid-derived suppressor cells (MDSCs), and tumor-associated macrophages (TAMs) with an M2-like phenotype. More specifically, the accumulation of MDSCs represents a major contributor to the poor immunotherapy response of MSS CRC, as these cells suppress cytotoxic T-cell activation, produce immunosuppressive cytokines such as TGF-β, promote Treg expansion, and interfere with the maturation and function of antigen-presenting dendritic cells (DCs). Similarly, TAMs further amplify local immunosuppression through extracellular matrix remodeling and promotion of neoangiogenesis. Collectively, these immunosuppressive myeloid and stromal components are strongly associated with poor clinical outcomes and resistance to immunotherapy [22]. Moreover, another hallmark of this phenotype is (v) the limited activation of interferon-mediated and other inflammatory signaling pathways. As a result, MSS/pMMR CRC is commonly characterized as a “non-inflamed” or “immune-cold” tumor microenvironment (TME) [23]. Additionally, (vi) the reduced expression of immune checkpoint molecules further contributes to the limited responsiveness of these tumors to immune checkpoint inhibitors (ICIs) [16,17,18,19,20,21,22,23].

Beyond their low mutational and neoantigen burden, MSS/pMMR tumors exhibit multiple complex mechanisms that collectively promote an immunosuppressive TME and facilitate immune evasion. Major resistance mechanisms include the activation of several oncogenic signaling pathways, such as transforming growth factor-β (TGF-β), WNT/β-catenin, PI3K/AKT, and MAPK signaling [23]. Among these, TGF-β-mediated stromal exclusion represents one of the most prominent mechanisms. Overexpression of TGF-β promotes the activation of cancer-associated fibroblasts (CAFs) and extensive stromal remodeling, resulting in the formation of a dense extracellular matrix enriched with fibronectin and collagen fibers. However, TGF-β not only induces desmoplasia, which limits the infiltration of cytotoxic T cells into the TME and impairs effective anti-tumor immune responses, but also suppresses T-cell proliferation and inhibits the maturation of antigen-presenting DCs. Furthermore, TGF-β amplifies the immunosuppressive milieu of the TME by promoting the polarization of tumor-associated macrophages (TAMs) toward the M2 phenotype, as well as the expansion of Tregs [16,17,18,19,20,21,22,23].

Furthermore, activation of the oncogenic WNT/β-catenin signaling pathway represents another major resistance mechanism, primarily driven by *APC* gene mutations, leading to impaired antigen presentation through reduced dendritic cell chemotaxis and infiltration within the tumor microenvironment (TME). This phenomenon is largely attributed to the absence or downregulation of key chemokines within the TME, such as CCL4, which is crucial for dendritic cell recruitment, resulting in ineffective tumor immune surveillance and facilitating immune escape [24].

In addition, mutations involved in the activation of other oncogenic pathways, such as MAPK (including *BRAF*, *KRAS*, and *NRAS*), promote the secretion of immune checkpoint molecules and contribute to TME enrichment with immunosuppressive immune cells and cytokines. Under these conditions, MHC machinery is also compromised, as MHC class I expression is often reduced, thereby impairing effective antigen presentation and promoting inefficient cytotoxic T-cell-mediated antitumor activity [25].

Alongside the overexpression of TGF-β, VEGF is also found at elevated levels within the TME of MSS colorectal cancers CRCs. The immunosuppressive effect of overexpressed VEGF in TME constitutes another resistance mechanism in MSS-CRC, as it not only promotes neoangiogenesis but also impairs the maturation of DCs and the antigen presentation mechanism, leading to tumor escape. More specifically, the newly formed abnormal vasculature, acts as a barrier for the infiltration and trafficking of immune cells. However, it promotes the expression of immunosuppressive molecules, as well as the expansion of several immunosuppressive cell populations such as TAMs, MDSCs, and Tregs. In this context, VEGF can serve as a druggable target for MSS-CRC via the utilization of anti-VEGF agents [26].

### 2.3. Exception of the Dichotomy Between MSS/pMMR and MSI-H/dMMR-CRC

Nevertheless, it should be emphasized that the dichotomous classification of CRC into MSI-H and MSS tumors is not definitive. Interestingly, a subset of MSS tumors can either exhibit an ultra-high TMB, or (ii) can harbor POLE/POLD1 exonuclease-domain mutations, presenting an increased amount of neoantigens, and an “inflamed” TME, with anti-tumor effector cells infiltrates. Despite being classified as MSS, these tumors have several similarities with MSI-H/dMMR tumors, as they exhibit sensitivity to immune checkpoint blockade. This phenomenon underscores the significance of multi-parameter integration, such as TMB and genomic instability status, which should be taken into consideration for patient risk stratification and therapy planning [17,23,27].

In Table 1, we present the differences between these two phenotypes [15,16,17,18,19,20,21,22,23,24,25,26].

## 3. Differential Responses to ICIs in MSI-H/dMMR and MSS/pMMR CRC

The clinical efficacy of ICIs is strongly influenced by microsatellite status. MSI-H/dMMR tumors generally demonstrate robust and durable responses to ICIs, largely due to their “inflamed” or “hot” TME. In contrast, MSS/pMMR tumors are relatively unresponsive and largely refractory to immunotherapy because of their low immunogenicity and the presence of multiple resistance mechanisms that impair antitumor immune surveillance. Consequently, microsatellite phenotype has emerged as a critical biomarker for predicting immunotherapy responsiveness and guiding treatment decisions, with ICIs currently representing the standard-of-care (SOC) therapy for metastatic MSI-H/dMMR CRC. In contrast, most immunotherapeutic strategies for MSS/pMMR CRC remain investigational, and they will be discussed in the following sections [28,29].

### 3.1. ICIs in Metastatic MSI-H/dMMR CRC

#### 3.1.1. Single-Agent PD-1 Blockade (Pembrolizumab) in Metastatic MSI-H/dMMR CRC

The randomized phase III KEYNOTE-177 trial compared pembrolizumab to standard chemotherapy as first-line therapy for unresectable or metastatic MSI-H/dMMR CRC. Pembrolizumab significantly prolonged progression- free survival versus chemotherapy (median PFS ≈ 16.5 vs. ≈8.2 months) and demonstrated a favorable safety profile and durable responses. These results supported pembrolizumab as a standard first-line option for metastatic MSI-H/dMMR CRC [30].

#### 3.1.2. Combinational Immune Checkpoint Blockade (Nivolumab ± Ipilimumab) in Metastatic MSI-H/dMMR CRC

Nivolumab alone and combined with low-dose ipilimumab in MSI-H/dMMR metastatic CRC was evaluated in the CheckMate-142 trial. This combination achieved high objective response rates and durable disease control, including in patients previously treated with chemotherapy. Long-term follow-up confirmed sustained benefits and an acceptable safety profile, supporting its use in selected patients and further investigation in both first-line and later settings [31,32].

Regulatory agencies recognized the survival advantage and tolerability of checkpoint blockade in MSI-H CRC. Pembrolizumab received Food and Drug Administration (FDA) approval for first-line treatment of unresectable or metastatic MSI-H/dMMR CRC following KEYNOTE-177 [30], and nivolumab (with or without ipilimumab depending on setting) is endorsed in guideline algorithms for previously treated MSI-H disease [31,32]. These approvals and guideline changes emphasize the necessity of early MSI/MMR testing to identify candidates for immunotherapy.

### 3.2. ICIs in Metastatic MSS/pMMR-CRC

In contrast to MSI-H/dMMR CRC, for which ICIs have been established as SOC, there is no ICI for patients with the MSS/pMMR phenotype. A substantial body of research has shown that patients with MSS/pMMR mCRC do not benefit from immune checkpoint inhibitor (ICI) monotherapy [33,34]. The limited efficacy of single-agent ICIs in this subgroup is primarily driven by low neoantigen burden, poor T-cell infiltration, and a highly immunosuppressive tumor microenvironment (TME). Furthermore, tumor-intrinsic signaling pathways, including signaling pathway alterations, together with stromal components such as dense extracellular matrix deposition (e.g., collagen) and CAFs activity, contribute to impaired antigen presentation and restricted T-cell trafficking, as previously described in Section 2. Collectively, these mechanisms represent major barriers to effective antitumor immunity, reinforcing the concept that MSS CRCs are poorly responsive to immunotherapy in their native state. Several combinatorial therapeutic strategies with ICI have been tested in order to improve the immunogenic tumor microenvironment and augment the efficacy of ICI in metastatic CRC patients with pMMR/MSS phenotype [35]. As a result, current therapeutic strategies for MSS CRC are largely focused on converting “cold” tumors into “hot” immunologically active tumors or on combining checkpoint blockade with agents that modulate tumor biology, vascular function, or antigen presentation to enhance immune responsiveness [36,37,38,39]. Consequently, current treatment strategies rely primarily on conventional chemotherapy, targeted therapies, and enrollment in clinical trials evaluating novel immunotherapy-based combinations [40,41,42].

In Table 2, standard-of-care (SOC) and Guideline-supported options for MSI-H/dMMR CRC [30,31,32].

## 4. Therapeutic Strategies to Enhance Immunotherapy Efficacy in MSS/pMMR CRC-Cold-to-Hot Concept

Given the limited efficacy of ICI monotherapy in MSS/pMMR CRC, current therapeutic approaches aim to transform these immune-cold tumors into immunologically active, or “hot,” tumors. This strategy aims at antigen presentation enhancement, reducing local immunosuppression, and promoting T-cell infiltration. Extensive Preclinical and Clinical Work Aims to Sensitize MSS CRC to Immunotherapy. Some of the key strategies include several drug combinations such as (i) ICI plus chemotherapy, (ii) Double Checkpoint Inhibitory Strategy (e.g., botensilimab plus balstilimab), (iii) ICI + targeted therapy (e.g., anti-angiogenic, etc.) or radiotherapy, (iv) therapeutic vaccines and (v) adoptive cell therapies, as well as (vi) bispecific T-cell engagers (BiTEs), and (vii) other checkpoint inhibitors, in combinational blockade [40,41,42,43,44,45,46,47,48,49,50,51,52,53,54,55,56].

### 4.1. ICI Plus Chemotherapy

Chemotherapy induces immunogenic cell death, increases antigen release, and transiently reduces immunosuppressive cells. Several trials are exploring ICI combination with cytotoxic regimens (e.g., FOLFOX, FOLFIRI) in metastatic and neoadjuvant settings. The results have been mixed, while some cohorts show enhanced immune infiltration or modest responses. However, the large-scale efficacy of their combination has to be further established [40,41,42].

### 4.2. Double Checkpoint Inhibitory Strategy

The combination of botensilimab (novel Fc-enhanced anti-CTLA-4 antibody), with balstilimab (anti-PD-1 antibody), constitutes a promising immunotherapeutic approach for the treatment of MSS-CRC, a subtype typically resistant to immune checkpoint blockade.

Preclinical and early-phase clinical data suggest that botensilimab enhances innate and adaptive immune activation, beyond the effects of conventional CTLA-4 inhibitors, potentially overcoming immune resistance in MSS CRC tumors [43].

Moreover, the combination of botensilimab and balstilimab has demonstrated encouraging antitumor activity in a phase 1b clinical trial, producing durable responses in heavily pretreated patients with MSS CRC, while maintaining a manageable safety profile.

Notably, the objective response rates and progression-free survival (PFS) observed in this cohort exceeded historical benchmarks reported for immunotherapy in MSS CRC, suggesting a potentially important advance in this traditionally treatment-resistant population. The enhanced Fc-effector functions of botensilimab may facilitate greater engagement of myeloid and NK cell pathways, thereby contributing to its broader immunomodulatory effects [44].

Further randomized studies are currently underway to validate these findings and identify optimal biomarkers for patient selection. In particular, the phase 2 trial NCT05608044 is evaluating the combination of botensilimab and balstilimab in patients with MSS mCRC without liver metastases.

Similarly, subgroup analyses from the phase 1b expansion study (NCT03860272) involving patients with non-liver metastatic MSS CRC demonstrated clinical responses across multiple metastatic sites, including the lungs, with activity also observed in patients with lung-only disease [43,44].

However, the FDA has advised against pursuing an accelerated approval pathway for this combination in relapsed or refractory MSS CRC, indicating that the currently available efficacy data are not yet sufficient to support regulatory approval. Nevertheless, if these findings are confirmed in larger randomized trials, this regimen could substantially expand the role of immunotherapy in MSS CRC and address a major unmet clinical need [43,44].

### 4.3. ICI + Anti-Angiogenic Agents

Bevacizumab and other anti-VEGF drugs can normalize tumor vasculature and reprogram myeloid compartments to be less suppressive, potentially synergizing with ICIs. Early clinical combinations and translational analyses suggest biologic plausibility; ongoing randomized trials are assessing clinical benefit [45].

### 4.4. ICI + Targeted Pathway Inhibitors

MEK inhibitors, EGFR inhibitors, or other targeted drugs may modulate tumor-intrinsic immune evasion mechanisms. For example, MEK inhibition can enhance antigen presentation and T-cell infiltration in preclinical models; combination clinical trials are ongoing, though tolerability and optimal sequencing/dosing remain challenges [46].

### 4.5. ICI + Radiation Therapy

Radiation induces DNA damage and immunogenic cell death, releasing tumor antigens and pro-inflammatory cytokines; small studies have reported abscopal effects (responses at distant, non-irradiated sites) when combined with ICIs, and larger trials are exploring this strategy in oligometastatic and metastatic CRC [47].

### 4.6. Vaccines, Oncolytic Viruses, and Adoptive Cell Therapy

Personalized neoantigen vaccines, viral oncolytic (which lyse tumor cells and stimulate innate/adaptive immunity), and adoptive cell therapies (tumor-infiltrating lymphocytes, TCR-engineered T cells, CAR T cells targeting shared CRC antigens) are in early-phase development [48]. These strategies aim to generate or amplify antigen-specific T-cell responses within MSS tumors [49].

### 4.7. Bispecific Antibodies and Other Immune Modulators

Furthermore, pivotal cellular functions and division have been suppressed using small therapeutic agents, which are associated with adverse events of varying grades, some of which significantly impact the patient’s overall well-being. Nevertheless, newer targeted strategies, such as engineered antibody-based therapy, present fewer side effects [50]. These agents bind to specific epitopes on the surface of malignant cells [51].

Other promising therapeutic agents include agonists for costimulatory receptors (OX40, 4-1BB), bispecific T-cell engagers (BiTEs), and other checkpoint inhibitors (LAG-3, TIM-3), which, in combination with PD-1 blockers, constitute alternative approaches to activating anti-tumor responses and are evaluated in ongoing trials [52,53]. An example of a promising T-cell BsAb is cibisatamab, which binds to the CD3 and CEA neoantigens, located on T cells and the malignant cell surface, respectively [54,55]. This agent has shown promising anti-tumor activity in preclinical disease models by activating PD-L1 and PD-1 and increasing the recruitment and infiltration of T-cells within the tumor [54]. At the same time, when combined with PD-1/PD-L1 inhibitors, it induces malignant cell death in vitro [55]. An example of the aforementioned combination is cibisatamab (sustained-dose escalation) and atezolizumab for metastatic MSS- CRC, which has been evaluated for safety in an ongoing phase I clinical trial for early-stage CRC [56]. Another ongoing phase I trial has also used cibisatamab as monotherapy [56]. Despite the promising effects and anti-tumor activity of BsAbs, their use remains challenging because tumors and the TME can be heterogeneous, and the anti-tumor immune response, including T-cell activation, can be suboptimal. The adverse effects could also be considered challenges, as could the demand for continuous administration [56]. Lastly, several biomarkers have been studied for the identification and optimal selection of CRC patients’ treatment. Among them, CEA constitutes a frequently overexpressed biomarker (over 80% of CRC cases) in the malignant tissue. Despite extensive research, a therapeutic combination that can reproduce the high response rates seen in MSI- H tumors in most MSS CRC cases has not yet been realized.

Finally, most combination approaches in MSS CRC remain investigational and have not yet demonstrated broad practice-changing efficacy. In Table 3, we summarize the therapeutic strategies for MSS/pMMR CRC tumors, which are still under evaluation and development.

## 5. Beyond MSI: Future Biomarkers of ICI Responsiveness in CRC Toward Precision Immunotherapy

### 5.1. Clinically Validated Biomarkers

MSI-H phenotype is the strongest single predictor of ICI responsiveness in CRC, with the clinical practice recommendation, as demonstrated in Table 4 [57].

### 5.2. Emerging Biomarkers

#### 5.2.1. TMB

Elevated TMB has been associated with increased tumor immunogenicity and improved responsiveness to ICIs in several tumor types, primarily through neoantigen generation that can be recognized by the anti-tumor immune system. However, the predictive value of TMB in CRC remains context-dependent and is influenced by several factors such as cause of hypermutation, microsatellite status, and the biological background of the tumor [58,59,60].

Evidence supporting the clinical relevance of TMB was demonstrated in the KEYNOTE-158 trial, in which pembrolizumab was administered to previously treated CRC patients with advanced or unresectable disease. In this study, patients with TMB ≥ 10 mutations per Mega-base (mut/Mb) showed a higher objective response rate (ORR) of 29%, compared with an ORR of 6% among those with TMB < 10 mut/Mb. Based on these findings, the FDA approved pembrolizumab for the treatment of high TMB (≥10 mut/Mb) unresectable, advanced solid tumors, or with refractory disease following prior treatment [61,62].

Moreover, pMMR/MSS CRC tumors with high TMB had a more favorable outcome after disease progression on Regorafenib/nivolumab therapeutic regime, based on the results of the REGONIVO trial. As it was shown, patients with CRC tumors (≥28 mts/Mb), who received combinational dual immunotherapy, had a higher OS, in comparison to those with TMB < 28 mts/Mb [63,64].

Even though TMB-high status is mostly seen in MSI-H/dMMR CRC tumors, a subset of MSS/pMMR tumors that present POLE or POLD1 exonuclease-domain mutations is characterized by an ultra-mutated phenotype, with a high load of neoantigens, and increased infiltrate of cytotoxic cells, despite the microsatellite stability [65].

#### 5.2.2. Polymerase ε and Polymerase δ (POLE/POLD1) Mutations

POLE and POLD1 genes encode the major catalytic and proofreading subunits of the Polε (polymerase epsilon) and Polδ (polymerase delta) enzyme complexes, respectively [66]. Mutations in these genes can impair DNA repair mechanisms, leading to increased TMB and enhanced neoantigen presentation within the TME. These alterations have been associated with improved responses to immunotherapy across multiple cancer types, highlighting their potential as emerging pan-cancer biomarkers [65,66,67].

In CRC tumors bearing mutated POLE/POLD1 are usually pMMR/MSS but show higher CD8^+^ T-cell infiltration and increased number of PD-1/PD-L1 and CTLA-4 protein expression, resembling MSI-H tumors and potentially responding to checkpoint inhibitors [17]. However, such mutations occur in less than 1% of colorectal cancer patients. Several clinical studies established that pembrolizumab and combinations like durvalumab with avelumab may benefit mCRC patients with MSS phenotype and POLE/POLD1 mutations. This benefit appears strongest among patients with POLE exonuclease domain mutations tumors and higher TMB, while other mutations may not lead to immunotherapy sensitivity [62].

#### 5.2.3. PD-L1 Expression

PD-1 contains an extracellular IgV-like domain, a hydrophobic transmembrane region, and a cytoplasmic tail with ITIM and ITSM motifs that transmit inhibitory signals to activate immunosuppression in TME. PD-L1 is structurally similar to PD-1, more conserved, and more widely expressed than PD-L2, playing a key role in tumor immune evasion. Antibodies against PD-1/PD-L1 have been approved by the FDA for the treatment of several cancer types, marking a new age in immunotherapy [68]. Solid tumors with elevated PD-L1 expression are often associated with better responses to ICIs [69]. However, increased PD-L1 levels are not a reliable predictor of treatment response in CRC and do not show a clear association with MSI-H/dMMR status [70,71].

Ongoing research is further examining the relationship between PD-L1 expression, MSI/MMR status, and clinical outcomes [72]. In contrast to several other malignancies, PD-L1 expression in CRC has shown limited and inconsistent predictive value. Although PD-L1 is used as a predictive biomarker in other gastrointestinal cancers, such as gastric and esophageal tumors, current evidence does not support its use as a predictive biomarker in mCRC patients with a pMMR/MSS phenotype. Another single-arm, multicenter Phase II AVETUX trial evaluating avelumab in combination with cetuximab and mFOLFOX6 in previously untreated RAS/BRAF wild-type, MSS mCRC patients also did not establish any association between PD-L1 expression and PFS [73].

#### 5.2.4. Circulating Tumor DNA(ctDNA) and Immunotherapy Response

Cell-free DNA (cfDNA) constitutes a dynamic, non-invasive molecular approach that has expanded the possibilities for detecting and analyzing extracellular DNA fragments across various biological samples. In oncology, it enables downstream identification of genetic and epigenetic aberrations without the need for tissue biopsy, through the analysis of circulating tumor DNA (ctDNA), which represents a tumor-derived subset of cfDNA. CtDNA refers to small fragments of DNA derived from cancer cells circulating in the bloodstream, released through tumor turnover, apoptosis, or direct secretion. These fragments may carry genomic alterations, including those related to MSI status, as well as other genetic and epigenetic aberrations. Its dynamic nature, reflecting the tumor’s genetic profile in real time, highlights its emerging role as a non-invasive “liquid biopsy” marker in several research settings [74].

This biomarker is primarily used for prognostic assessment and treatment monitoring, although there is still insufficient evidence to support its use as a predictor or guide of response to ICIs. In the context of response monitoring, ctDNA clearance is generally associated with treatment response, whereas persistence or an increase indicates residual disease or therapeutic resistance. Meanwhile, a prognostic marker, higher baseline levels are associated with worse PFS and OS, while post-treatment persistence correlates with an increased risk of disease recurrence [74,75].

In addition, ctDNA may enable early detection of subclinical disease or minimal residual disease (MRD), as it can identify microscopic disease prior to radiological evidence. Nevertheless, it remains an unvalidated predictive biomarker for ICI treatment planning and is currently used mainly as a monitoring tool, either alone or in combination with other biomarkers such as MSI status, TMB, and tumor microenvironment features. CtDNA has increasingly been recognized as both a prognostic and predictive biomarker in CRC. Multiple studies have reported an association between ctDNA levels and chemotherapy response in patients with mCRC [74,75].

Its potential clinical value is also being investigated in patients with locally advanced rectal cancer (LARC) receiving neoadjuvant chemoradiotherapy (nCRT). Baseline preoperative ctDNA levels have been linked to pathological TNM stage and tumor response; however, they are not yet reliable for accurately predicting complete response (CR). Longitudinal monitoring of ctDNA appears promising for patient risk stratification. For instance, a baseline mutant variant allele frequency (mVAF) of ≥0.5% or detectable ctDNA during nCRT has been associated with worse PFS and overall survival (OS). Additionally, integrating mVAF, TMB, and ctDNA clearance can categorize patients into high- and low-risk groups with significantly different survival outcomes [76,77]. Combining ctDNA analysis with other data sources, including genomic, epigenetic, TME, transcriptomic, and radiomic information, may enhance the accuracy of complete response assessment. Furthermore, the presence of preoperative ctDNA and high-risk gene mutations is increasingly considered when identifying patients who may not be appropriate candidates for a watch-and-wait management approach [78], while dynamic ctDNA clearance during immunotherapy may reflect treatment response or resistance.

Last but not least, emerging approaches such as methylation-based analyses and fragmentomics may further improve disease detection and treatment response assessment, while longitudinal ctDNA profiling offers the potential for real-time monitoring of tumor dynamics, including disease progression and the development of acquired resistance during immunotherapy [79].

#### 5.2.5. Microbiome Composition

Gut microbiome plays a crucial role in the development and maintenance of the host immune system, and the efficacy of checkpoint inhibitors in the treatment of tumors is related to gut microbiota [80]. There is growing evidence that the interaction between the intestinal microbiota and the host immune system can significantly modulate antineoplastic immune responses. Microbiome-associated immune modulation can influence not only local antitumor responses but also systemic immunity, thereby contributing to CRC progression. It has been demonstrated that specific bacterial species within the intestinal microbiome, such as *Fusobacterium nucleatum*, are implicated in the suppression of cytotoxic immune cells, the enhancement of immunosuppressive cell recruitment, and the activation of multiple inflammatory pathways. In contrast, beneficial commensal bacteria can promote dendritic cell recruitment, enhance T-cell-mediated antitumor activity, and increase sensitivity to immune checkpoint blockade. Meanwhile, microbial products such as bile acid derivatives and other metabolites also modulate immune responses, opening new horizons for microbiome-targeted therapeutic strategies, including fecal microbiota transplantation and probiotic or prebiotic administration, with the aim of enhancing immunotherapy efficacy [81].

A study has shown that the ratio of Prevotella to Bacteroides in the gut of patients with gastrointestinal tumors is associated with the efficacy of ICIs, and whether they can benefit from immunotherapy is likely to be related to the metabolites of the bacteria [82]. Another study highlighted that a microbiome containing 11 different types of bacteria collected from the human gut was able to trigger CD8+ T-cells capable of producing IFN-γ in mouse models, thereby enhancing the anti-tumor efficacy of ICIs [83], while it has been found that Clostridium in the baseline intestinal microflora could be used as a putative biomarker to predict the response of ICIs [82,84]. In addition, it has been reported that in non-CRC tumors treated with ICIs, antibiotic use may alter the number and function of immune cells in the gut, and alterations in the gut microbiota may negatively affect immunotherapy efficacy [81,82,83,84]. Emerging evidence suggests that intestinal microbes modulate systemic antitumor immunity and may influence responsiveness to ICIs. Integration of multi-omic data (genomics, transcriptomics, proteomics, microbiome) in prospective trials is essential to develop composite predictive tools for immunotherapy in CRC [81,82,83,84].

## 6. Emerging and Future Directions in CRC Treatment Management

Early clinical trials have highlighted remarkable tumor regressions with neoadjuvant PD-1 blockade in MSI-H/dMMR colorectal cancer (CRC), prompting the exploration of organ preservation and treatment de-escalation strategies [85].

Personalized immunotherapies, including neo-antigen vaccines and T-cell therapies, aim to augment immune responses to patient-specific neoantigens and may extend benefit beyond MSI-H tumors, including tumors with MSS status [86]. Given the well-known role of the gut microbiome in modulating immunotherapy responses, interventions such as fecal microbiota transplantation and microbial metabolite modulation are under investigation to increase ICI sensitivity [85,86,87]. Another active direction involves rational combination strategies that pair ICIs with targeted agents or novel immunomodulators, including inhibitors of TGF-β, adenosine pathways, myeloid cells (CSF-1R), or metabolic pathways [88,89]. Finally, integration of ctDNA-based liquid biopsies for minimal residual disease (MRD) detection offers a precision-oncology approach to guide adjuvant immunotherapy and inform early treatment escalation or de-escalation decisions [75].

Last but not least, emerging technologies, including biosensor-based platforms, may further increase the real-time detection of novel circulating biomarkers, enhance their sensitivity, and enable not only the identification of the disease in early stages, but also the monitoring and the tailored-made therapeutic decision-making [90].

## 7. Conclusions

Microsatellite phenotype is a central biomarker in CRC that guides immunotherapy administration. MSI-H/dMMR patients show strong and often durable responses to PD-1-based therapies (pembrolizumab, nivolumab ± ipilimumab), which are currently approved even in the first-line setting for metastatic disease. On the other hand, MSS/pMMR CRC is largely resistant to the single-ICI strategy, and overcoming this resistance remains a key challenge. Current research is therefore focused on combination strategies, as well as the development of novel modalities. For this reason, universal MSI/MMR testing is essential for both treatment selection and familial risk assessment. Ultimately, MSS/pMMR CRC resistance to ICIs requires a deeper understanding of the resistance mechanisms, as well as the development of biomarker-guided therapeutic strategies to expand immunotherapy benefit to this highly frequent population.

## Figures and Tables

**Table 1:** Microsatellite Instability-High (MSI-H) vs. Microsatellite Stable (MSS) Colorectal Cancer.

Feature	MSI-H/dMMR (Immune-Hot)	MSS/pMMR (Immune-Cold)
TMB/neoantigens	↑ TMB ↑ neoantigen load	↓ TMB ↓ neoantigens
Immune infiltration	↑ Dense CD8^+^ T cells, NK cells, Th1 cells	↓ Poor effector T-cell infiltration enriched suppressive cells, MDSCs, Tregs, TAMs (M2)
Inflammation	Active IFN-γ signaling, CXCL9/10 production	↓
Immune checkpoints	↑ PD-1/PD-L1 expression	↓
TME profile	Inflamed, immune-active	Immunosuppressive, immune-cold
Main resistance mechanisms	↑ T-cell exhaustion, B2M/JAK mutations, immunoediting (in subsets)	↑ WNT/β-catenin, MAPK, TGF-β signaling, stromal exclusion, VEGF-driven suppression
Key suppressive factors	↑ MDSCs, Tregs, TAMs (M2)	↑ Desmoplasia, MDSCs, Tregs, TAMs (M2)
Response to ICIs	↑ Strong	↓ Poor

B2M, β2-microglobulin; CRC, colorectal cancer; dMMR, deficient mismatch repair; ICIs, immune checkpoint inhibitors; IFN-γ, interferon-gamma; JAK, Janus kinase; MAPK, mitogen-activated protein kinase; MDSCs, myeloid-derived suppressor cells; MSS, microsatellite stable; MSI-H, high microsatellite instability; NK, natural killer; PD-1, programmed cell death protein 1; PD-L1, programmed death-ligand 1; pMMR, proficient mismatch repair; TAMs, tumor-associated macrophages; TGF-β, transforming growth factor-beta; TMB, tumor mutational burden; TME, tumor microenvironment; Tregs, regulatory T cells; VEGF, vascular endothelial growth factor; WNT, Wingless/Integrated signaling pathway; ↑, high/increased; ↓, low/reduced.

**Table 2:** Standard-of-care (SOC) and Guideline-supported options for MSI-H/dMMR CRC.

Category	Therapeutic Strategy	Examples	Current Status in MSI-H/dMMR CRC	Actions/Limitations
**MSI-H/dMMR CRC**				
**SOC**	PD-1 blockade	Pembrolizumab	Approved first-line treatment for metastatic MSI-H/dMMR CRC	Durable responses due to high TMB, abundant neoantigens, and inflamed TME
**Guideline-supported options**	PD-1 ± CTLA-4 blockade	Nivolumab ± Ipilimumab	Recommended for mCRC in selected cases	Enhances T-cell activation and improves response durability

CRC, colorectal cancer; dMMR, deficient mismatch repair; ICIs, immune checkpoint inhibitors; MSI-H, high microsatellite instability; PD-1, programmed cell death protein 1; PD-L1, programmed death-ligand 1; SOC, standard-of-care; TMB, tumor mutational burden.

**Table 3:** Emerging strategies to enhance immunotherapy responsiveness in MSS/pMMR colorectal cancer [40,41,42,43,44,45,46,47,48,49,50,51,52,53,54,55,56].

Strategy	Approaches	Development Stage	Evidence	Main Objective
ICI + Chemotherapy	FOLFOX/FOLFIRI + PD-1/PD-L1 inhibitors	Clinical	Mixed	Enhance antigen release and immune activation
Dual Checkpoint Blockade	Botensilimab + Balstilimab	Clinical	Engouraging	Simultaneous activation of innate and adaptive immunity
ICI + Anti-Angiogenic Therapy	Bevacizumab-, Regorafenib-, or Fruquintinib-based combinations	Clinical	Promising	Normalize vasculature and reduce immune suppression
ICI + Targeted Therapy	MEK, EGFR, or TGF-β inhibitors	Clinical		Overcome tumor-intrinsic immune resistance
ICI + Radiotherapy	PD-1/PD-L1 inhibitor combinations	Clinical		Promote antigen release and immune priming
Vaccines and Oncolytic Viruses	Neoantigen vaccines, viral therapies	Early clinical development	Exploratory	Generate tumor-specific immune responses
Adoptive Cell Therapy	CAR-T cells, TCR-engineered T cells, TILs	Early clinical development		Enhance antigen-specific T-cell activity
Bispecific Antibodies (BiTEs)	Cibisatamab and related agents	Early clinical development		Redirect T cells toward tumor cells
Other Immune Modulators	OX40, 4-1BB, LAG-3, TIM-3 targeting agents	Early clinical development		Augment anti-tumor immune responses

**Table 4:** Clinical implications of microsatellite phenotype in colorectal cancer management [57].

Clinical Setting	Indications	Recommendations	Key Considerations
Universal MSI/MMR Testing	All newly diagnosed CRC cases	MMR immunohistochemistry (IHC) and/or PCR/NGS-based MSI testing	Identifies MSI-H/dMMR tumors and supports Lynch syndrome screening
Stage II MSI-H/dMMR CRC	Localized disease	Individualized adjuvant treatment decisions	Favorable prognosis; limited benefit from adjuvant 5-FU-based chemotherapy
Metastatic MSI-H/dMMR CRC	Advanced disease	First-line PD-1 blockade (e.g., pembrolizumab); nivolumab ± ipilimumab in selected cases	Potential for durable responses; monitor immune-related adverse events
Metastatic MSS/pMMR CRC	Advanced disease	Prioritize clinical trial enrollment	Standard chemotherapy and targeted therapies remain the backbone of treatment
Lynch Syndrome	Suspected or confirmed hereditary disease	Genetic counseling and surveillance programs	Family screening, risk assessment, and cancer prevention

CRC, colorectal cancer; MSI-H, high microsatellite instability; dMMR, deficient mismatch repair; MSS, microsatellite stable; pMMR, proficient mismatch repair; PD-1, programmed cell death protein 1; PD-L1, programmed death-ligand 1; ICIs, immune checkpoint inhibitors.

## Data Availability

No new data were created or analyzed in this study. Data sharing is not applicable to this article.

## Abbreviations

APM	Antigen Presentation Machinery
B2M	β2-microglobulin
BiTEs	Bispecific T-cell Engagers
CAFs	Cancer-Associated Fibroblasts
CAR-T	Chimeric Antigen Receptor T cells
CEA	Carcinoembryonic Antigen
CIMP	CpG Island Methylator Phenotype
CRC	Colorectal Cancer
CTLA-4	Cytotoxic T-Lymphocyte-Associated Protein 4
ctDNA	Circulating Tumor DNA
DCs	Dendritic Cells
dMMR	Deficient Mismatch Repair
DNA	Deoxyribonucleic Acid
EGFR	Epidermal Growth Factor Receptor
FDA	Food and Drug Administration
FOLFIRI	Folinic Acid + Fluorouracil + Irinotecan
FOLFOX	Folinic Acid + Fluorouracil + Oxaliplatin
HLA	Human Leukocyte Antigen
ICI/ICIs	Immune Checkpoint Inhibitor(s)
IFN-γ	Interferon-gamma
ITIM	Immunoreceptor Tyrosine-based Inhibitory Motif
ITSM	Immunoreceptor Tyrosine-based Switch Motif
JAK	Janus Kinase
LAG-3	Lymphocyte Activation Gene-3
MAPK	Mitogen-Activated Protein Kinase
mCRC	Metastatic Colorectal Cancer
MDSCs	Myeloid-Derived Suppressor Cells
MHC-I	Major Histocompatibility Complex Class I
MHC-II	Major Histocompatibility Complex Class II
MLH1	MutL Homolog 1
MMR	Mismatch Repair
